# Granulocyte colony-stimulating factor (G-CSF) increases histone-complexed DNA plasma levels in healthy volunteers

**DOI:** 10.1007/s10238-016-0413-6

**Published:** 2016-03-22

**Authors:** Christian Schoergenhofer, Michael Schwameis, Philipp Wohlfarth, Christine Brostjan, Simon T. Abrams, Cheng-Hock Toh, Bernd Jilma

**Affiliations:** 1 Department of Clinical Pharmacology, Medical University of Vienna, Währinger Gürtel 18-20, 1090 Vienna, Austria; 2 Department of Internal Medicine I, Medical University of Vienna, Vienna, Austria; 3 Department of Surgery, Medical University of Vienna, Vienna, Austria; 4 Institute of Infection and Global Health, University of Liverpool, Liverpool, UK

**Keywords:** G-CSF, Histones, NETs, Gender, Neutrophils

## Abstract

Granulocyte colony-stimulating factor (G-CSF) is an activator of neutrophil granulocytes. Neutrophil extracellular traps are a defensive mechanism consisting of neutrophils, platelets, DNA, histones and antimicrobial proteins. This study was performed to determine whether G-CSF increases histone-complexed DNA in the plasma of healthy volunteers. In total, 51 healthy volunteers (25 males and 26 females) were treated with G-CSF (18 with 300 µg single dose i.v., 27 with 5 µg/kg s.c. for 4 days) and six participants received a placebo. Histone-complexed DNA was measured by enzyme immunoassay in plasma samples at predefined time points (0, 2, 4, 6, 24 h after single dose, day 1, day 2 and day 5 after repeated doses). Histone levels were quantified by Western blotting. A single dose of G-CSF rapidly increased hc-DNA by about 50 % (*p* < 0.05 for 2–24 h). After repeated doses the increase was even more pronounced: hc-DNA increased by about 50 % (3.0 ± 0.9, *p* < 0.001 after 24 h and about fourfold after 96 h (*p* < 0.001)). A statistical significant increase in histone levels was detected as early as 4 h after G-CSF injection (0.43 ± 0.2 vs. 1.08 ± 0.3 µg/ml; *p* = 0.034). In the placebo group no significant changes occurred. Moreover, significantly higher levels of hc-DNA were measured in male compared to female subjects (226 ± 43 vs. 84 ± 19, *p* < 0.001). G-CSF injection substantially increases hc-DNA levels in healthy volunteers. There is a significant gender difference in hc-DNA at the baseline.

## Introduction

Granulocyte colony-stimulating factor (G-CSF) not only stimulates the growth and proliferation of hematopoietic progenitor cells, but also activates mature granulocytes. Binding of G-CSF to its receptor prolongs the lifespan of granulocytes, enhances the phagocytic activity and induces degranulation of preformed and stored defensive substances and modulators of the immune system [1–3]. Endogenous plasma levels of G-CSF rise during infections, experimental endotoxemia [4, 5] or neutropenia [6].

Recently a new defensive mechanism was discovered. In a process called “NETosis” neutrophil extracellular traps (NETs) are formed. In short, stimulation of neutrophils leads to an interaction with the endothelium and to chromatin decondensation by enzymes (i.e., neutrophil elastase, myeloperoxidase or peptidyl arginine deiminase type IV). Neutrophils then release decondensated DNA, associated antimicrobial factors and histones. These “traps” are ready to catch and attack pathogens [7]. Hence, histone-complexed DNA (hc-DNA) increases in the blood of patients suffering from severe sepsis [8]. Also erythrocytes and blood platelets are associated with NETs in blood vessels. As NETs also seem to play a pathogenic role in deep venous thrombosis, they may constitute an important link between inflammation and thrombosis [9, 10]. G-CSF injection markedly enhances platelet aggregation and shear-dependent platelet function [11, 12].

In a mouse model investigating the prothrombotic state of cancer patients the involvement of G-CSF in NET formation was demonstrated for the first time [13]. Thus, we hypothesized that injection of G-CSF may increase plasma levels of hc-DNA in healthy volunteers.

## Materials and methods

### Study design

Written informed consent was obtained from all study participants before study entry. The study protocol was approved by the Independent Ethics Committee of the Medical University of Vienna, and the trial was registered in a public database (EudraCT No. 2006-005582-18, 2008-004550-32). The study was conducted in accordance with the good clinical practice guidelines and the Declaration of Helsinki. The trial took place at the Medical University of Vienna, Department of Clinical Pharmacology, and was conducted between December 8, 2006, and June 17, 2009.

Healthy men and women between 18 and 55 years, with a body weight ≤96 kg, were included in the study. All participants signed an informed consent form before any trial-related activity was performed. All female subjects with childbearing potential performed a negative urine pregnancy test before inclusion. All subjects were nonsmokers with normal laboratory values and normal findings in their medical history.

Exclusion criteria contained known spleen enlargement, hypersensitivity to any of the formulations’ ingredients, clinically relevant abnormal laboratory values, use of any medication during 2 weeks before the start of the study, a clinically relevant illness within 3 weeks before the start of the study, a recent infection within 1 week, skin abnormalities at the injection site or any other medical condition deemed relevant by the principal investigator.

The trial took place at the Department of Clinical Pharmacology of the Medical University of Vienna. The trial was performed to investigate biosimilarity between two G-CSF formulations. In the trial protocol, storage of plasma samples for academic purposes was planned before the initiation of the trial. In one part of the project, healthy volunteers received 300 µg of G-CSF intravenously (i.v.). In another part, a double blind, randomized, active and placebo-controlled, parallel group trial, healthy volunteers were randomized to receive 5 µg/kg G-CSF subcutaneously or a placebo on four consecutive days. This dose was chosen with regard to the primary endpoint of the trials, the absolute neutrophil count. Increasing the G-CSF dose to 10 µg/kg bodyweight does not have a major influence on the absolute neutrophil count [14], but increases the amount of CD34+ cells in peripheral blood. However, CD34+ cells were only a secondary endpoint in these trials. Thus, we decided not to further increase the dose to reduce potential hazards to healthy volunteers. Furthermore, this dose should be sufficient to activate peripheral neutrophils.

### Blood sampling

Samples were drawn using intravenous catheters placed in an appropriate antecubital vein. After discarding the first 2 mm of blood, blood was drawn into sterile blood tubes anticoagulated with 3.8 % sodium citrate or EDTA (© Vacuette). Additionally intravenous catheters were flushed with 0.9 % sodium chloride solution after each sampling time point. Blood samples were drawn before administration and at predefined time points thereafter (0, 2, 4, 6, 24 h and days 1, 2 and 5). Blood samples were then centrifuged at 2000*g* at 4 °C for 15 min. Plasma was stored at −80 °C until analyzed.

### Biomarker assays

We performed enzyme immunoassays to detect blood levels of hc-DNA. Cell death detection ELISA plus kit (©Roche) uses monoclonal mouse antibodies against histones (antihistone-biotin, clone H11-4) and single- and double-stranded DNA (anti-DNA POD, cloneMCA-33) to detect apoptotic or necrotic cells in plasma or cell culture supernatants. In short, histone-complexed mono- and oligonucleosomes are quantified. The presented values are light absorbance values (405 nm) directly derived from the described assay and therefore arbitrary units. The assay was performed according to the manufacturers’ instructions. Histone levels were quantified by Western blotting using antihistone H3 antibodies (Abcam) and calculated using human recombinant histone H3 as standard, as described previously [15]. We performed these tests in a smaller number of samples in collaboration with the University of Liverpool. Measurements were taken in samples of five randomly chosen patients of each study.

### Statistical analysis

All data are expressed as mean ± standard error of the mean (SEM) unless otherwise stated. For reasons of robustness we have chosen nonparametric tests. Within-group differences were tested overall by Friedman ANOVA, and pairwise comparisons were made by Wilcoxon signed rank test. Results of histone quantification were compared by pairwise *T* tests after logarithmic transformation. Nonparametric tests were not applied because of the small sample size. Where applicable, baseline data were compared with the nonparametric Kruskal–Wallis test, followed by a *U* test. Correlations were performed by the nonparametric Spearman test. A two-tailed *p* < 0.05 was considered statistically significant. All statistical calculations were performed using commercially available statistical software (IBM SPSS Statistics version 22).

## Results

### Baseline data

We analyzed samples of six participants receiving a placebo, 27 participants treated with 5 µg/kg body weight for four consecutive days and 18 participants receiving a single dose of 300 µg G-CSF intravenously.

Baseline characteristics of participants of each study are presented in Table 1. No significant differences can be found between the placebo group and the G-CSF groups with regard to height, weight, body mass index, neutrophil counts, platelet counts or the level of C-reactive protein.

**Table 1:** Presented are demographic data of all study participants (mean ± SD)

Studies	300 µg i.v. G-CSF	5 µg/kg G-CSF	Placebo	*p* value
*Baseline data*				
Gender m/f	9/9	15/12	1/5	0.32
Age (years)	30 ± 2	29 ± 1	26 ± 2	0.57
Height (cm)	171 ± 2	173 ± 2	168 ± 3	0.34
Weight (kg)	69 ± 3	70 ± 2	63 ± 7	0.43
Body mass index (kg/m^2^)	23.8 ± 0.8	23.3 ± 0.8	22 ± 1.6	0.76
Neutrophil count (G/L)	3.2 ± 0.3	3.0 ± 0.2	3.5 ± 0.2	0.28
Platelet count (G/L)	228 ± 12	229 ± 11	244 ± 17	0.62
C-reactive protein (mg/dl)	0.2 ± 0.1	0.4 ± 0.2	0.2 ± 0.1	0.50

No significant differences were found between groups

All values are presented as mean ± SEM, and *p* values are the results of nonparametric tests

### Histone-complexed DNA

A single dose of 300 µg G-CSF significantly increased hc-DNA in the plasma of healthy volunteers 2, 4, 6 and 24 h after the intravenous injection (*p* = 0.003 for overall testing with Friedman ANOVA; compared to baseline, Table 2 with pairwise comparison). Significantly higher levels of hc-DNA were found at each time point after the G-CSF administration compared to the baseline levels. However, after 2 h there was no further increase in the amount of circulating hc-DNA.

**Table 2:** Levels of histone-complexed DNA in the plasma of study participants before and 2, 4, 6 and 24 h after a single intravenous injection of G-CSF are presented

	Mean	SEM	IQR	*p* value
Baseline	197	32	69–299	
2 h	290	43	171–357	0.006*
4 h	287	46	133–357	0.02*
6 h	293	35	142–435	0.001*
24 h	292	50	126–484	0.006*

Values presented resemble baseline values and time after G-CSF administration

*SEM* standard error of the mean, *IQR* interquartile range

* Wilcoxon tests

Subcutaneous injection of 5 µg/kg G-CSF time dependently increased plasma levels of hc-DNA after 24 and 96 h compared to the baseline (*p* < 0.001 for overall testing with Friedman ANOVA, compared to baseline, Table 3 with pairwise comparison), and the increase after 96 h was more pronounced (*p* < 0.001; 24 vs. 96 h).

**Table 3:** Levels of histone-complexed DNA after four consecutive daily doses of 5 µg/kg body weight and the results of the placebo group

	Mean	SEM	IQR	*p* value
0 h	152	41	30–194	
*Placebo*	34	10	20–54	
24 h	231	51	74–272	<0.001
*Placebo*	77	37	23–59	0.4
96 h	598	97	255–867	<0.001
*Placebo*	55	20	15–94	0.6

Values resemble baseline values (0 h) and time after daily G-CSF doses. *p* values are the results of nonparametric tests versus baseline values

*SEM* standard error of the mean, *IQR* interquartile range

In the placebo group no significant changes in any of the investigated parameters could be detected (*P* = 0.85) (Table 3).

### Histone quantification

At the baseline we measured total histone levels of 0.43 ± 0.20 µg/ml, which increased to 1.71 ± 0.54 µg/ml (*p* = 0.088) 2 h and 1.08 ± 0.30 µg/ml (*p* = 0.034) 4 h after infusion of 300 µg G-CSF.

In the study investigating effects of four daily doses of G-CSF we detected 1.39 ± 0.37 µg/ml, 24 h after the first G-CSF injection 1.24 ± 0.26 µg/ml and on day five, 24 h after the fourth injection of 5 µg/kg G-CSF, 2.16 ± 0.62 µg/ml. There was no significant difference between these measurements. However, a borderline increase between days two and five (*p* = 0.064) was found.

### Gender differences

To examine potential gender differences we performed a pooled analysis of blood samples taken at the baseline. Samples of 26 female and 25 male healthy volunteers were analyzed. We detected significantly higher amounts of hc-DNA in male subjects than in female subjects (226 ± 43 in male vs. 84 ± 19 in female subjects, *p* < 0.001).

**Fig. 1:**
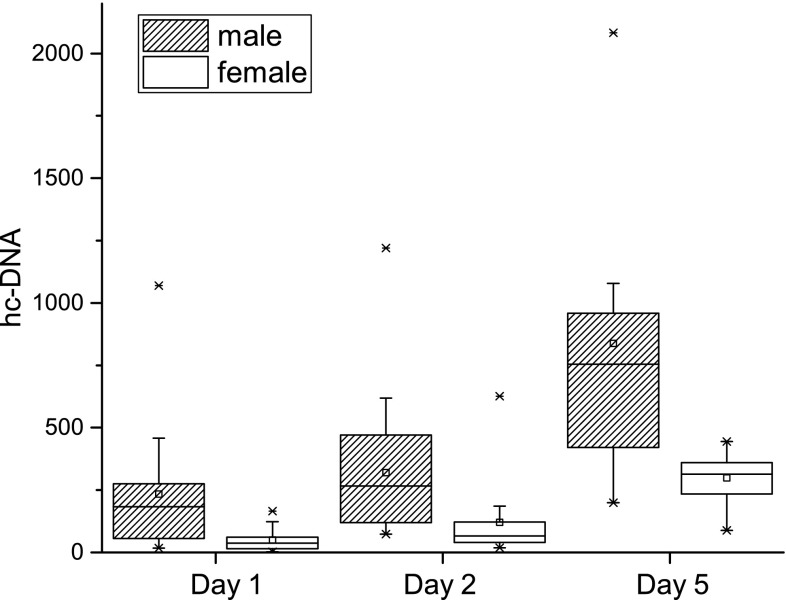
Gender differences in histone-complexed DNA before and after 4 daily doses of 5 µg/kg body G-CSF. Although in male and in female subjects a significant increase in hc-DNA after single (day 2) or multiple (day 5) doses of G-CSF can be detected, a significant gender difference can be identified. Female subjects have significantly lower hc-DNA levels at the baseline, on day 2 and on day 5, compared to men. Presented data are means ± SEM

The sample size of the subgroup receiving a single dose was probably too limited to demonstrate significant gender differences between nine male and nine female volunteers (232 ± 46 vs. 161 ± 42, *p* = 0.34). In contrast, the trial investigating daily doses of 5 µg/kg G-CSF demonstrated marked gender differences at all times (*p* < 0.002; Fig. 1; *n* = 15 male and 12 female subjects).

Within female subjects levels of hc-DNA rose from baseline values of 50 ± 14 to 120 ± 48 and 299 ± 29 after 24 and 96 h (*p* = 0.012, and *p* = 0.002, respectively), whereas in male subjects baseline values of 234 ± 67 rose to 319 ± 76 and 838 ± 148 after 24 and 96 h (*p* = 0.005 and *p* = 0.001, respectively) (Fig. 1).

### Correlations

**Fig. 2:**
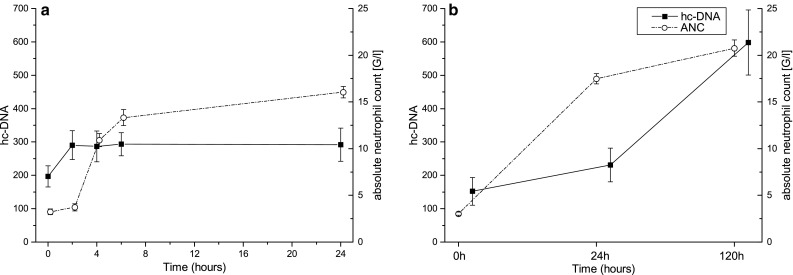
**a** Time course of hc-DNA and ANC after 300 µg G-CSF single dose, correlation coefficient 0.272, **b** time course of hc-DNA and ANC after daily 5 µg/kg G-CSF, correlation coefficient 0.310. The correlation between hc-DNA and neutrophil granulocyte count over time is small. This indicates that the level of plasma hc-DNA is not depending on the neutrophil count, but may be more dependent on the activation of neutrophil granulocytes. Presented are means ± SEM

Although both hc-DNA and neutrophils increased over time, an exploratory correlation analysis including all measured values revealed only a weak correlation within each study. In the single dose study the correlation coefficient was 0.272 (*p* = 0.009), and in the study of multiple doses the correlation coefficient was 0.310 (*p* = 0.005). Figure 2 shows that there is a time lag between the increase in neutrophils with almost a plateau after 24 h and the maximum levels of hc-DNA.

## Discussion

This trial demonstrates (1) that infusion of a single dose of 300 µg G-CSF significantly increases hc-DNA in the plasma of healthy volunteers, (2) that this increase is even more pronounced after injection of four daily doses of G-CSF, (3) that there is a significant difference in serum levels of hc-DNA between male and female subjects, at the baseline and after injection of G-CSF, and (4) histone plasma levels significantly increase as early as 4 h after a single G-CSF injection.

Neutrophils of mice receiving daily G-CSF as well as neutrophils of mice bearing G-CSF-producing cancer cells are predisposed to generate NETs after activation [13]. Our findings support these results and underline the involvement of G-CSF in hc-DNA release in humans. Additionally we were able to show that a single dose of G-CSF is sufficient to rapidly increase levels of hc-DNA in plasma.

Levels of G-CSF are elevated during infections [6]. Usually this is explained by the need for a greater number of neutrophils, and infection-associated leukocytosis is the result of this increase. However, among its specific roles, the cell-activating properties of G-CSF are of special interest in this study. Binding of G-CSF to its receptor leads to internalization of the receptor and to an immediate activation of neutrophils. Consecutively activated neutrophils release the contents of their preformed granules and leave the blood stream into the surrounding tissue. An immediate but transient drop in the absolute neutrophil count in peripheral blood can be observed shortly after injection of G-CSF [3, 17].

A recently discovered defensive mechanism of neutrophil granulocytes called “NETosis” is described as a cell death distinct from apoptosis and necrosis. NETosis is a cell death program that results in the expulsion of decondensated DNA from neutrophil granulocytes alongside the exertion of other preformed substances stored in various granules within the cell. This DNA builds loose congregates of neutrophils, platelets, erythrocytes, and antimicrobial substances as well histones that entangle the pathogens and possibly kill them [7, 18]. Interestingly, *Staphylococcus aureus* is able to produce enzymes, which degrade these NETs and escape this defensive mechanism [19].

In this study we measured hc-DNA before and after G-CSF administration. Although we did not further identify the origin of this circulating extracellular DNA, it seems likely that the DNA is of neutrophil origin. An increase in plasma myeloperoxidase after G-CSF treatment was reported previously. This supports our hypothesis, as this enzyme is not just of neutrophil origin, but can also be found in NETs [7, 20]. The absolute neutrophil count rises after G-CSF injection. However, the correlation between the neutrophil count and the DNA overall was rather weak (correlation coefficient 0.310 and 0.272, respectively). This indicates that the amount of hc-DNA in the plasma is not merely depending on the number of circulating neutrophils itself, but on the number of activated neutrophils. Our results of the substudy investigating the effect of a single intravenous dose of 300 µg/kg G-CSF underline this observation. Two hours after injection we found a significant increase in hc-DNA although neutrophil numbers did not rise notably. In contrast, 24 h after this single dose, the amount of hc-DNA did not further increase compared with the level at 2 h post-dose, although the number of circulating neutrophils rose almost fivefold (Fig. 2). This is in accordance with several studies demonstrating rapid activation of preformed neutrophils after G-CSF administration [3, 21].

Abrams et al. pointed out that histones play a pathophysiological role in patients suffering from trauma, but also from necrotizing pancreatitis or sepsis. In their mouse model, infusion of histones led to an increase in thrombin–antithrombin complexes indicating activation of coagulation. Furthermore in 52 trauma patients circulating histone levels were correlated with sequential organ failure assessment (SOFA) scores [15]. In our study, all measured histone levels were low and within the normal range of healthy volunteers. Abrams et al. reported median histone levels of 2.3 µg/ml in healthy volunteers, but 28.6 µg/ml in trauma patients. The increases in our study were small, and statistical findings may also be due to the small sample size. On the other hand G-CSF injection did also not lead to a relevant activation of blood coagulation (see below). Moreover, repeated doses of G-CSF increased C-reactive protein levels approximately twofold (0.23–0.51 mg/dl). However, this increase is still within the limits of the normal range [12]. Considering these findings, the increase in hc-DNA may be more likely to be a consequence of an increase in circulating DNA rather than an increase in histones.

Beside neutrophils, the activation of the endothelium and endothelial damage may be the source of hc-DNA. G-CSF injection also leads to endothelial activation as demonstrated by Stroncek et al. However, in their study they administered 10 µg/kg G-CSF for 5 days and showed an increase in E-selectin of 78 ± 34 % after 5 days [22]. A similar regimen was used in another study that also demonstrated increases of E-selectin after 5 days [20]. Thus, the pronounced increase of hc-DNA on day five may also be attributable to endothelial activation. On the other hand, no data exist yet investigating immediate effects of a single G-CSF dose on the endothelium. Finally, we cannot exclude that both, the endothelium and neutrophils, may contribute to that rise, which is a limitation of this trial.

Interestingly, there was a significant difference in the levels of hc-DNA between male and female healthy volunteers, which is in contrast to the results of Tillack et al. [23]. In their work, Tillack et al. detected gender differences in patients diagnosed with remittent-relapsing multiple sclerosis, but not in healthy volunteers or any other patient group under investigation. We found significantly higher levels of plasma hc-DNA in male than in female subjects in the pooled analysis of baseline values (26 females and 25 males) and within the substudy of healthy volunteers receiving consecutive G-CSF doses.

The role and importance of NETs and circulating extracellular DNA in the pathophysiology of thrombosis are currently intensively investigated [24–26]. A single dose of G-CSF increases von Willebrand factor activity slowly by ~60 % which may in part contribute to accelerated platelet plug formation under high shear rates [11]. While the effects of G-CSF on platelet aggregation are pronounced and clear-cut, the G-CSF-induced increase in coagulation appears to be limited in vivo. Multiple doses of G-CSF enhance platelet aggregation [12]. Despite a transient > fivefold increase in whole-blood TF-mRNA 4 h after a single G-CSF dose, and a somewhat accelerated clotting time in rotational thromboelastometry, G-CSF increases prothrombin fragment levels only mildly (~30 %) and transiently in the blood of healthy volunteers [11]. Furthermore G-CSF at doses of 10 µg/kg body weight for 5 days also increased the tissue factor-dependent coagulation [27]. In striking contrast, D-dimer levels do not change after G-CSF in healthy volunteers [11, 12], arguing against relevant induction of clotting in vivo. Thus, the G-CSF-enhanced release of hc-DNA does not go along with a clinically relevant extent of in vivo thrombin generation. It was proposed that the main driver for thrombin generation may be the histone content of NETs and that these histones need to be dismantled, i.e., by the addition of DNAse, to trigger thrombin generation [16]. This was also demonstrated for endothelial damage, as intact nucleosomes do not exert toxic effects. When these nucleosomes were degraded, by incubation with serum or by sonication, toxic effects on endothelial cells were detected [28]. Thus, it is conceivable that G-CSF may lead to NET formation, but histones within NETs remain shielded and no relevant induction of clotting results.

Furthermore, usually only healthy people before stem cell harvest or patients during neutropenia, which is frequently accompanied by thrombocytopenia, are treated with G-CSF. Thus, thrombotic events are generally rare and more of episodic character. A large trial investigating adverse events in peripheral blood stem cell donors found no increased risk of thrombosis after mobilization with G-CSF compared to bone marrow donors without stem cell mobilization [29]. Finally, pattern recognition receptors for extracellular DNA have been identified and C-reactive protein levels are known to rise after multiple doses of G-CSF in healthy volunteers [12, 30]. Thus, the increased hc-DNA levels may contribute to the activation of the inflammatory cascade after treatment with G-CSF. To what extent hc-DNA contributes to this inflammatory response remains unclear due to its complexity and needs to be further elucidated by additional studies.

## Conclusion

Injection of single and multiple doses of G-CSF increased levels of hc-DNA significantly. Interestingly, a gender difference with lower levels of hc-DNA in female than in male subjects was detected.
